# Basolateral amygdala volume in affective disorders using 7T MRI *in vivo*

**DOI:** 10.3389/fpsyt.2024.1404594

**Published:** 2025-01-06

**Authors:** Benedikt Kürzinger, Stephanie Schindler, Martin Meffert, Anja Rosenhahn, Robert Trampel, Robert Turner, Peter Schoenknecht

**Affiliations:** ^1^ Department of Psychiatry and Psychotherapy, University Hospital Leipzig, Leipzig, Germany; ^2^ Department of Neurophysics, Max Planck Institute for Human Cognitive and Brain Sciences, Leipzig, Germany; ^3^ Out-patient Department for Sexual-therapeutic Prevention and Forensic Psychiatry, University Hospital Leipzig, Leipzig, Germany; ^4^ Department of Psychiatry, Psychotherapy and Psychosomatic, Saxon State Hospital Altscherbitz, Schkeuditz, Germany

**Keywords:** amygdala, basolateral amygdala, volume, major depressive disorder, FreeSurfer, BLA, MDD, 7T

## Abstract

**Background:**

The basolateral complex of the amygdala is a crucial neurobiological site for Pavlovian conditioning. Investigations into volumetric alterations of the basolateral amygdala in individuals with major depressive disorder (MDD) have yielded conflicting results. These may be reconciled in an inverted U-shape allostatic growth trajectory. This hypothesized trajectory unfolds with an initial phase of volumetric expansion, driven by enhanced dendritic arborization and synaptic plasticity. The increase in volume is followed by a reduction phase, as glucocorticoid exposure cumulatively results in excitotoxic damage, reflecting allostatic load.

**Methods:**

7T magnetic resonance brain imaging was conducted on a total of 84 participants (mean age 38 ± 12 years), comprising 20 unmedicated and 20 medicated individuals with MDD, 21 individuals suffering from bipolar disorder and 23 healthy controls. We employed FreeSurfer 7.3.2 for automatic high-resolution segmentation of nine amygdala subnuclei. We conducted analyses of covariance, with volumes of the basolateral complex, the lateral nucleus and, exploratively, the whole amygdala, as dependent variables, while controlling for the total intracranial volume and sex. Quadratic regressions were computed within the MDD group and in relevant subgroups to investigate the presence of a U-shaped relationship between the number of preceding major depressive episodes or the duration of the disease since the first episode and the dependent variables.

**Results:**

Diagnostic groups did not exhibit statistically significant differences in the volumes of the basolateral amygdala (left *F* (3,75) = 0.66, *p* >.05; right *F* (3,76) = 1.80, *p* >.05), the lateral nucleus (left *F* (3,75) = 1.22, *p* >.05; right *F* (3,76) = 2.30, *p* >.05)), or the whole amygdala (left *F* (3,75) = 0.48, *p* >.05; right *F* (3,76) = 1.58, *p* >.05). No quadratic associations were observed between surrogate parameters of disease progression and any of the examined amygdala volumes. There were no significant correlations between subregion volumes and clinical characteristics.

**Conclusion:**

We found no evidence for the hypothesis of an inverted U-shaped volumetric trajectory of the basolateral amygdala in MDD. Future research with larger sample sizes, including the measurement of genetic and epigenetic markers, will hopefully further elucidate this compelling paradigm.

## 1 Introduction

Affective disorders comprise major depressive disorder (MDD), also known as unipolar affective disorder, and bipolar disorder (BP). Affective disorders are mental diseases with high prevalence, inflicting severe consequences on the affected individuals and contributing greatly to the global burden of disease (1). For example, MDD is estimated to affect about one in every six adults during their lifespan (2). It is a heterogeneous disease with diverse manifestations (3–6).

As mood, anxiety and stress are inherently interconnected (7, 8), a foundational understanding in contemporary psychiatry is that mood disorders stem from prolonged stress responses, leading to dysregulation of the hypothalamic-pituitary-adrenal (HPA) axis (9, 10). A meta-analysis examining HPA-axis dysregulation in MDD detected medium to small elevations in adrenocorticotropic hormone and cortisol and a reduction in corticotropin releasing factor (CRF) levels (11).

The amygdala is a subcortical brain twin structure, located bilaterally in the anterior medial temporal lobe (12), consisting of 15 nuclei (13). Its medial and central nuclei are projecting to the paraventricular nuclei of the hypothalamus, e.g., via the bed nucleus of the stria terminalis (14), rendering it an important part of the HPA axis. Amygdala activation enhances hypothalamic CRF secretion (15).

Drawing upon cytoarchitectural and functional assessments, the amygdala is frequently categorized into three primary subdivisions: the basolateral, centromedial, and superficial amygdala (16, 17). The basolateral complex of the amygdala (BLA) is the main input site, receiving information from all sensory systems (visual, auditory, somatosensory, olfactory, and gustatory), from the hippocampus and the entorhinal cortex, and from polymodal association cortices (18). It is composed of the lateral nucleus (LA), the basal nucleus (also referred to as the basolateral nucleus) and the accessory basal nucleus (often also named basomedial nucleus). The neuronal morphology of the BLA resembles that of the neocortex and is comprised mostly of glutamatergic pyramidal neurons which express multipolar dendritic trees that are covered with spines. Their axons form numerous projections to other BLA neurons, amygdala nuclei, or more remote brain areas (19).

The amygdala is crucial for evaluating the emotional significance of incoming stimuli (20). It mediates appropriate physiological reactions (e.g., autonomic reactivity), memory consolidation (via reciprocal connections with the hippocampus) and behavioral adjustments (such as reward processing and modulation of social behavior, via reciprocal connections with the ventromedial prefrontal cortex and posterior orbitofrontal cortex) (18, 21, 22). Neuroimaging studies in healthy individuals have shown that the amygdala is particularly activated during processing of negative emotions, most predominantly fear (23). There is evidence from multimodal MRI studies that the amygdala is crucially involved in the pathophysiology of depression. Enhanced glucose metabolism and heightened resting cerebral blood flow in the amygdala have been shown for individuals affected by MDD (24, 25). Decreased connectivity of the frontal lobe to the amygdala (26) is thought to lead to an increased activity of the amygdala in depressed patients. Increased amygdala activation in the face of a negative stimulus in depressed individuals has been reported in a meta-analysis (27).

The BLA is a pivotal locus for associative learning, central in encoding environmental cues, contexts, and behaviors, thereby delineating the boundary between safety and recognized threats (28). Associative learning unfolds as the brain establishes connections among previously disparate elements, such as objects, sights, sounds, ideas, or behavior. This process, known as conditioning, intertwines the significance of one stimulus with that of another (29). Synaptic plasticity in excitatory and inhibitory circuits in the BLA, especially in the LA, have been well established as the cellular substrate of Pavlovian associative learning (30, 31). Pavlovian associative learning describes the triggering of physiological and behavioral changes in response to a conditioned, initially neutral stimulus, by the use of an aversive, unconditioned stimulus (32). It is required for humans and other mammals to scan and respond to their environment (32) and is a near-ideal model to identify processes involved in fear acquisition and extinction (33, 34).

On the pyramidal neurons of the LA, highly processed input from sensory cortices converges with direct subcortical inputs via the thalamus (22, 35). In more detail, excitatory synaptic inputs conveying conditioned (e.g., sound) stimuli and unconditioned stimuli (e.g., electric shock) converge on the same pyramidal neuron in the LA, leading to NMDA receptor-mediated long-term potentiation conveying the conditioned stimulus to the LA (Hebbian plasticity) (36). This leads to a strengthening of the synapses carrying the conditioned stimulus information (37, 38). While the central amygdala also contributes to fear conditioning (19), our study focused on the BLA and the LA. Their neurocircuitries provide a complex yet fairly well understood model to approximate the real-world phenomena of fear and threat learning.

The BLA is known to express mineralocorticoid and glucocorticoid receptors, which are both susceptible to glucocorticoids (39). Animal models employing chronic immobility stress to mimic depression have revealed elevated levels of glucocorticoids in the BLA (40, 41). After application of this paradigm, enhanced dendritic length, branching and spines in the BLA were observed (42, 43). Glucocorticoids are deemed to be the mediating factor of this link (40, 44). Spines are typically correlates of strongly excitatory synapses (28). Interestingly, the effects of glucocorticoids on the BLA seem to differ from their effects on other regions of the brain, e.g., the hippocampus, where dendritic retraction was observed after stress exposure (45, 46). Children with congenital adrenal hyperplasia exhibit reduced amygdala volumes (47) as well as patients with Cushing syndrome (48), underscoring the influence of elevated glucocorticoid levels on amygdala volumes. Glucocorticoids and CRF mediate changes of brain-derived neurotrophic factor (BDNF) levels by altering transcription of the BDNF genes (49). BDNF is a neurotrophin deemed to be potent modulator of neuronal survival, growth, and differentiation, and specifically regulates morphological plasticity of dendrites (50, 51). Lakshminarasimhan and Chattarji showed that chronic stress causes an up-regulation of BDNF in the BLA in rats (52). BDNF therefore is a potential pathway through which glucocorticoids influence dendritic-spine formation in the BLA (53). Antidepressant drugs as well have been proposed to act on amygdalar spines via BDNF (54–56).

Previous investigations into the subnuclei of the amygdala have produced inconsistent findings regarding the volume of the basolateral amygdala. Some studies reported volumetric increases (57), while others observed decreases (58, 59). Yet, there are also studies noting no discernible alterations (60–62). In patients with anxious MDD, which is one of the most common subtypes (63), Li et al. found no significant differences in amygdala subfield volume compared to patients with non-anxious MDD (64). A large sample morphometric study conducted by the ENIGMA group (65) revealed that patients experiencing their first MDD episode (*n* = 500) exhibited greater thickness and larger surface area in the BLA compared to those with recurrent episodes (*n* = 1,174). Additionally, this group observed that patients with an onset of MDD at or before age 21 (*n* = 476) had lower thickness and smaller surface area in the BLA relative to HC (*n* = 2,879). Post-mortem studies in humans by Rubinow et al. (66) revealed a larger lateral nucleus in depressed individuals and a higher total count of neurovascular cells in the BLA compared to controls, respectively. Notably, individuals with an MDD duration shorter than 5 years demonstrated a significantly higher number of neurovascular cells in the accessory basal nucleus compared to those with a longer history of the disease, suggesting time dependent effects of MDD on neurovascular cells. Rubinow et al. did not investigate whether the observed volume increase was specifically linked to enhanced dendritic arborization. Therefore, this augmentation may be attributed to dendritic branching, a higher count of neurovascular cells, or a combination of both factors.

The concept of allostatic load, as proposed by Danese and McEwen (67) may help to reconcile the contradictions observed in volumetric alterations. Allostatic load refers to the cumulative physiological burden placed on an organism as a result of prolonged exposure to elevated levels of stress, leading to structural adaptation processes. While these adaptations may promote short-term survival, they often fail to establish true homeostasis. Persistent exposure to stressors can result in chronic overactivation of the stress-responsive system, which is detrimental to the organism in the long term. In this context, histoanatomical allostatic adaptations in the amygdala are correlates of efforts to contextualize stimuli associated with threat and vigilance.

Hanson and Nacewicz (28) proposed an inverted-U shaped allostatic growth trajectory to explain changes in amygdala volume. They suggested that sustained stress initially leads to an increase in the volume of the BLA through enhanced dendritic arborization. This enlargement results in greater cellular complexity, particularly within excitatory synapses, leading to heightened excitation. However, as the balance between excitation and inhibition is disrupted, metabolic demands rise, causing the accumulation of neurotoxic compounds like glutamate. Glutamate, along with other excitatory amino acids, is known to have neurotoxic effects under certain conditions (68). Subsequently, the ensuing toxic-metabolic damage may lead to dendritic loss and volume reduction. Consistent with this, earlier studies provide evidence suggesting an increase in amygdalar volume in patients newly diagnosed with MDD (69, 70) followed by a decrease as the duration of MDD progresses (71).

Hypothesis I. Given that unmedicated depressed individuals (group MDDu) in our sample had a relatively short illness duration and few major depressive episodes (MDEs), we hypothesized that the volume of the basolateral amygdala (BLA) and its primary component, the lateral nucleus (LA), would be increased in these individuals compared to healthy controls as a consequence of allostatic adaptations (28).

Hypothesis II. For patients in the medicated depressed group (MDDm), characterized by longer illness durations and more MDEs compared to those in the MDDu group, we proposed that cumulative glucocorticoid exposure, leading to excitotoxic damage, would outweigh any neuroprotective effects of antidepressant medication. Consequently, we hypothesized volume reductions in the BLA and LA for the MDDm group compared to the MDDu group, even below the baseline volume observed in healthy controls (HC).

Hypothesis III. Considering the significance of the amygdala in anxiety disorders (72), we hypothesized that patients with comorbid anxiety disorders would exhibit smaller volumes of the BLA due to heightened activation and increased toxic-metabolic strain.

Hypotheses IV & V. Following the concept of allostatic growth trajectory, we hypothesized a negative quadratic correlation between the numbers of MDEs or the total duration of the disease and the volume of the BLA/LA in the major depressive disorder groups (MDDu and MDDm).

## 2 Materials and methods

### 2.1 Study design and participants

The study design incorporated four distinct groups, consisting of Caucasian in- and out-patients of the University Hospital of Leipzig. The ages ranged from 18 to 65 years, with a mean age of 38 ± 12 years (see **Table 1** for further information on the sample characteristics). Individuals with a history of substance dependency were strictly excluded.

**Table 1:** Comparison of sociodemographic and clinical characteristics of the study groups.

	Depression (unmedicated, *n* = 20)	Depression (medicated, *n* = 20)	Bipolar disorder (*n* = 21)	Controls (*n* = 23)	Group comparison	
					Test statistic	*p*-value
**Sex (male/female)**	8/12	7/13	9/12	9/14	*X^2^* (3, n = 84) = 0.27	.97
**Age**	36.2 ± 12.8	42.9 ± 10.8	39.3 ± 12.0	36.0 ± 12.8	*F* (3, 80) = 1.47	.23
**Laterality quotient**	90.2 (- 100.0-100.0)	100.0 (-30.0-100.0)	81.0 (-80.0-100.0)	100.0 (-77.8-100.0)	*X^2^ (3, n = 83) = 4.63 (k)*	.20
**Intracranial volume (liter)**	1.37 ± 0.15	1.36 ± 0.12	1.39 (1.17 - 2.04)	1.41 (1.14 - 2.16)	*X^2^* (3, n = 84) = 0.498 (k)	.92
**Body mass index**	25.7 ± 4.9	24.5 ± 6.1	25.0 ± 3.9	22.2 ± 2.1	*F* (3, 36) = 4.49 (w)	.009**
**Beck Depression Inventory II**	26.4 ± 10.2	21.6 ± 10.6	22.5 ± 13.4	n.a.	*F* (2, 58) = 0.97	.38
**Bech-Rafaelsen-Melancholia Scale**	16.3 ± 4.9	14.8 ± 7.9	18.6 ± 9.0	n.a.	*F* (2, 36) = 1.03 (w)	.37
**Hamilton Rating Scale for Depression**	17.5 ± 6.9	16.2 ± 9.7	19.5 ± 8.9	n.a.	*F (*2, 58*)* = 0.78	.46
**Inventory of Depressive Symptomology**	31.3 ± 12.4	30.9 ± 13.9	33.0 ± 15.9	n.a.	*F* (2, 58) = 0.13	.88
**Years since 1st episode**	7.6 ± 10.4	13.3 ± 10.5	15.9 ± 10.6	n.a.	*F* (2, 57) = 3.20	.048*
**Weeks since onset of current episode**	12.0 (3-100)	16.0 (2-29)	10.0 (2-56)	n.a.	*X^2^* (2, N = 58) = 0.60 (k)	0.74
**No. of depressive episodes**	2.0 (1-5)	4.5 (1-60)	8.0 (1-33)	n.a.	*X^2^* (2, N = 61) = 23.8 (k)	<.001***
**Overall no. of illness episodes**	2.0 (1-5)	4.5 (1-60)	11.0 (2-65)	n.a.	*X^2^* (2, N = 61) = 30.3 (k)	<.001***

Means ± SD or medians (range) for not normally distributed data, respectively, are listed. Degrees of freedom of the group comparisons (ANOVA or chi-squared, respectively) are given in brackets. Laterality quotient, as defined by the Edinburgh inventory (73). *p ≤.05, **p≤.01, ***p ≤.001, (k) KruskaI—WaIlis test, n.a, not available, (w) Welch Statistic.

Two groups of patients suffering from MDD were formed, all currently experiencing a depressed mood state. One of them, named MDDm, took medication at the time of the MRI scans. The second group, MDDu, consisted of patients who had abstained from psychopharmacological medication for a minimum of three months before undergoing 7T MRI. For each participant in the MDDu group, a healthy control (HC) matched in terms of sex, age and handedness was recruited. Most patients in the fourth group, which comprised individuals with bipolar disorder (BP), took medication. All participants provided written informed consent. The study was approved by the Ethics Committee of the University Leipzig, Germany, and carried out in accordance with the latest version of the Declaration of Helsinki.

Of 107 patients initially participating in the study, 101 underwent 3T MRI scanning to exclude neurological diseases. Structured Clinical Interview for DSM-IV (SCID) (74) assessments were conducted on 91 participants. Their disease severity was evaluated with self-rating scales such as the Beck Depression Inventory (BDI-II) (75), clinical rating scales like the Bech-Rafaelsen Melancholia Scale (BRMS) (76), and structured interviews including SIGH-D17/IDS-C3 (77) for the Hamilton Rating Scale for Depression (HRSD) and the Inventory of Depressive Symptomatology (IDS). We also recorded additional parameters including the duration of the disease, defined as the time interval between a patient’s current age and the age at first diagnosis, as well as the duration of the current MDE, and the number of MDEs (78) a patient had suffered from so far. 87 subjects completed the 7T MRI phase. After 2-4 years, axis I diagnoses were validated, resulting in a switch of diagnosis for one patient from MDD to BP. Ultimately, 84 individuals were included for volumetric analysis, with sample sizes of *n* = 20 for MDDm, *n* = 20 for MDDu, *n* = 23 for HC, and *n* = 21 for BP. The sample sizes ensured sufficient test power (1-β = 0.80) for large-sized effects with an alpha error rate of 5% in a one-way ANOVA with fixed effects and four groups (79). For further details please refer to Schindler et al. (78).

### 2.2 Image acquisition and pre-processing

A 7T whole-body MR scanner (MAGNETOM 7T, Siemens Healthineers, Erlangen, Germany) and a 24-channel NOVA coil (Nova Medical, Inc., Wilmington, MA, USA) were employed to acquire T1-weighted high (0.7 mm isotropic) resolution images of the brain.

A 3D Magnetization-Prepared 2 Rapid Acquisition Gradient Echoes sequence (MP2RAGE) (80) was used with the following parameters optimized for high contrast-to-noise ratio: repetition time (TR) = 8.25 s; inversion times (TI1/TI2) = 1 s/3.3 s; flip angles (a1/a2) = 7°/5°; echo time (TE) = 2.51 ms; and bandwidth (BW) = 240 Hz/Px, 1 average. A field of view (FOV) of 224 mm x 224 mm x 168 mm combined with an imaging matrix of 320 x 320 x 240 resulted in a nominal acquisition voxel size of 0.7 mm isotropic. With parallel imaging (81) and an acceleration factor of two, a scan time of 18:02 min could be achieved. The scans were performed from September 2010 to April 2014.

We used a combination of Medical Image Processing and Visualization software (MIPAV, version 7.0.1) (82), and Computational Anatomy Toolbox (CAT12; C. Gaser, Structural Brain Mapping Group, Jena University Hospital, Jena, Germany, http://www.neuro.uni-jena.de/cat/) to create binary brain masks. These masks contained information for every voxel, indicating whether it was categorized as brain tissues or meninges, or attributed to the skull or environment.

### 2.3 FreeSurfer segmentation

FreeSurfer image analysis suite (http://surfer.nmr.mgh.harvard.edu/) version 7.3.2 (August 2022) was used for cortical reconstruction and volumetric segmentation. FreeSurfer’s main and fully automatic image processing pipeline involves several steps such as motion correction and averaging of the T1 weighted image (83), removal of non-brain tissue using a hybrid watershed/surface deformation procedure (84), automated Talairach transformation, segmentation of subcortical structures (including the amygdala) (85, 86), and automated topology correction (87). FreeSurfer’s morphometric methods have been reported to exhibit strong reliability across different scanners and field strengths (88, 89).

**Figure 1:**
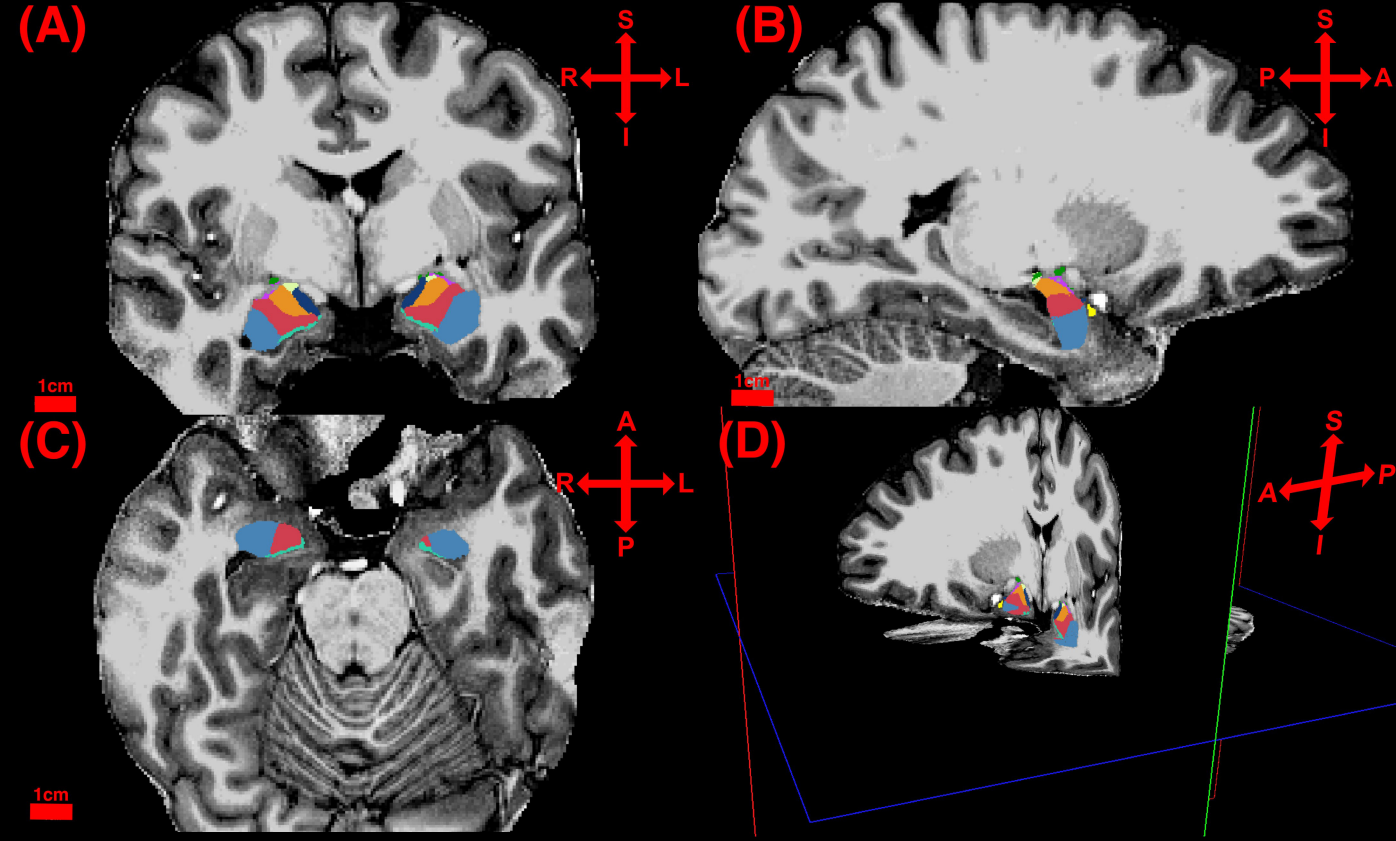
T1-weighted images at a resolution of 0.7 x 0.7 x 0.7 mm, depicting amygdala nuclei segmentation in coronal **(A)**, sagittal **(B)**, and axial **(C)** orientations, as well as in three-dimensional (3D) planar view **(D)**. Blue indicates the lateral nucleus, red the basal nucleus, and orange the accessory basal nucleus. A, Anterior; I, Inferior; L, Left; P, Posterior; R, Right; S, Superior.

The binary brain masks were input into the main pipeline to enhance FreeSurfer’s skull stripping process. For 20 images, the initially acquired NIfTI images required cropping of non-brain tissue to enable accurate Talairach transformation. Four subjects required manual white matter editing due to the failure of FreeSurfer’s automated topology correction. The editing was conducted in accordance with the official instructions as established by the FreeSurfer developers and available on their website. After completing FreeSurfer’s main pipeline, all segmentations were thoroughly visually quality-checked. Subsequently, the amygdala subfield segmentation (implemented in MATLAB runtime) (90) was performed (see **Figure 1**). Both the main pipeline and the subfield module generate estimates for total amygdala volumes. For subsequent analysis of total amygdala volume, the values obtained from the subfield module were selected due to their higher accuracy, as acknowledged by the FreeSurfer developers. Intracranial volume (ICV) was determined using FreeSurfer’s measurement of *estimated total intracranial volume*. Volumes of the left and right BLA were calculated by summing the computed volumes of lateral, basal, and accessory basal nuclei.

### 2.4 Data characteristics and statistical analyses

Statistical analyses were performed using SPSS Statistics 29 (SPSS Inc. Released 2022. PASW Statistics for Windows, Chicago, USA). All tests were two-tailed and *p* values below.05 were considered significant. Normality was tested using the Shapiro-Wilk test and confirmed for the volumes of total amygdala, BLA and LA as well as for age. Two subjects (study groups HC and BP) exhibited unusually high values for the ICV (|z| > 3.29). Visual inspection did not reveal major segmentation errors. Prior to further statistical analysis, these outliers were excluded from the dataset, resulting in confirmation of the assumption of normality. An additional individual in the BP group demonstrated unusually small left-sided volumes of interest (|z| for the left LA > 2.58, |z| for the left BLA > 3.29). As a result, this subject was excluded from the analysis of the left side only. Therefore, we proceeded with a sample size of *N* = 81 for the left-side volumes and *N* = 82 for the right-side volumes for further analysis.

As left and right-side volumes of interests highly correlated (whole amygdala and BLA *r* (83) = .90, LA *r* (83) = .88), a univariate approach was favored. Initially, a one-way ANOVA was performed for the left and right-side volumes each. The study groups were well balanced with regard to their main potential confounders ICV, sex, and age (see **Table 1**). The impact of these potential confounding variables on the dependent variables was examined as shown in **Table 2**. Based on these analyses, the group comparisons were repeated under stepwise inclusion of ICV and then sex (ANCOVA).

**Table 2:** Impact of confounding variables on volumes of interest.

Covariates	Left			Right		
	Whole Amygdala (*N* = 81)	Basolateral Complex (*N* = 81)	Lateral Nucleus (*N* = 81)	Whole Amygdala (*N* = 82)	Basolateral Complex (*N* = 82)	Lateral Nucleus (*N* = 82)
**ICV**	*r (*81*)* = .67, *p* <.001***	*r* (81) = .66, p <.001***	*r* (81) = .63, *p* <.001***	*r* (82) = .61, *p* <.001***	*r* (82) = .60, *p* <.001***	*r* (82) = .58, *p* <.001***
**Sex**	*t* (81)= 3.97, *p* <.001***	*t* (81) = 3.82, p <.001***	*t* (81)= 4.09, *p* <.001***	*t* (82)= 4.16, *p* <.001***	*t* (82) = 4.16, *p* <.001***	*t* (82) = 3.88, *p* <.001***
**Age**	*ρ* (81) = -.01, *p* =.915	*ρ* (81) = -.01, p =.941	*ρ* (81) = -.01, *p* =.932	*ρ* (82) = .09, *p* = .436	*ρ* (82) =.11, *p* =.323	*ρ* (82) = .12, *p* = .299

***p ≤.001, ICV: intracranial volume, Pearson’s correlation coefficient *r*, Spearman’s correlation coefficient *ρ*, Student’s *t* for independent t-tests. The *p* values listed in the tables are not corrected for multiple testing using Bonferroni correction.

To examine the disease progression-dependent inverted U-shaped trajectory, we conducted bivariate quadratic regressions between the number of MDEs and the volumes of the left and right BLA and LA for a pooled MDD group, as well as for the MDDu and MDDm group separately. Additionally, we computed bivariate quadratic regressions to explore the relationship between the overall duration of the disease since the onset of the first MDE and the volumes of interest for the same groups.

We assessed linear correlations using Spearman’s *rho* for non-normally distributed data and Kendall’s *τ* for non-equidistant ordinal data between the regions of interest and clinical characteristics, including age of onset, weeks since the onset of the current episode, BDI, BRMS, HRSD, IDS and the factor two (anxiety and arousal) items of IDS (91, 92). This assessment was conducted for the diagnostic groups separately, as well as for a pooled patients group (comprising BP, MDDu, and MDDm) and a pooled MDD group (comprising MDDu and MDDm).

## 3 Results

### 3.1 Group comparisons

In healthy controls, the absolute volume of the left amygdala was 1766.5 ± 206.4 mm³ and 1801.3 ± 199.9 mm³ for the right amygdala, 1380.2 ± 163.3 mm³ for the left BLA, and 1392.5 ± 156.4 mm³ for the right BLA. For the LA, volumes of 666.6 ± 78.5 mm³ (left) and 666.1 ± 70.9 mm³ (right) were obtained in healthy individuals. All volumes are presented in **Table 3**.

**Table 3:** Measured volumes in mm^3^ ± standard deviation and group comparisons.

	Healthy Controls (*n = 22)*	Depressed (unmedicated) (*n = 20)*	Depressed (medicated) (*n = 20)*	Bipolar disorder (*n(*Left) = 19, *n*(Right) = 20)	Group comparison	Corrected for ICV	Corrected for ICV & sex
**Left Whole Amygdala**	1766.5 ± 206.4	1763.0 ± 157.6	1739.4 ± 235.2	1690.1 ± 251.3	*F* (3,77) = 0.53, *p* = .663, partial *η^2^* = .020	*F* (3,76) = 0.53, *p* = .660, partial *η^2^* = .021	*F* (3,75) = 0.48, *p* = .694, partial *η^2^* =.019 **(H)**
**Left Basolateral Complex**	1380.2 ± 163.3	1375.1 ± 122.0	1359.6 ± 186.6	1314.3 ± 193.6	*F* (3,77) = 0.62, *p* = .602 partial *η^2^* = .024	*F* (3,76) = 0.66, *p* = .578, partial *η^2^* = .025	*F* (3,75) = 0.66, *p* =.582, partial *η^2^* = .026 **(H)**
**Left Lateral Nucleus**	666.6 ± 78.5	665.2 ± 71.6	660.5 ± 91.5	628.9 ± 88.6	*F* (3,77) = 0.90, *p* = .443, partial *η^2^* = .034	*F* (3,76) = 1.06, *p = .369*, partial *η^2^* = .040	*F* (3,75) = 1.22, *p* = .308, partial *η^2^* =.047
**Right Whole Amygdala**	1801.3 ± 199.9	1825.0 ± 158.5	1776.8 ± 258.4	1700.4 ± 266.5	*F* (3,78) = 1.17, *p* = .328, partial *η^2^* = .043	*F* (3,77) = 1.52, *p = .*215, partial *η^2^* = .056	*F* (3,76) = 1.58, *p* =.201, partial *η^2^* = .059
**Right Basolateral Complex**	1392.5 ± 156.4	1410.8 ± 116.6	1370.5 ± 205.7	1308.3 ± 202.2	*F* (3,78) = 1.33, *p* = .270, partial *η^2^* = .049	*F* (3,77) = 1.71, *p* = .172, partial *η^2^* = .062	*F* (3,76) = 1.80, *p* = .155, partial *η^2^* = .066
**Right Lateral Nucleus**	666.1 ± 70.9	675.1 ± 56.8	653.6 ± 104.3	622.02 ± 84.0	*F* (3,78) = 1.67, *p* = .181, partial *η^2^* = .060	*F* (3,77) = 2.12, *p* = .104, partial *η^2^* = .076	*F* (3,76) = 2.30, *p* = .084, partial *η^2^* = .083

ICV, intracranial volume. *F*: AN(C)OVA statistics, degrees of freedom are given in brackets. The *p* values listed in the table are not corrected for multiple testing using Bonferroni correction. (H) Homogeneity of regression slopes violated.

The study groups differed significantly in the number of MDEs and the time elapsed since the first MDE, but not in clinical depression severity ratings (see **Table 1**).

Stepwise global comparisons (ANOVA, ANCOVA with sex, and ANCOVA with sex and ICV) did not find significant differences between the study groups with respect to the mean volume of the BLA (e.g., full model left *F* (3,75) = 0.66; right *F* (3,76) = 1.80, all *p* >.05) or the mean volume of the LA (left *F* (3,75) = 1.22; right *F* (3,76) = 2.30, all *p* >.05). No significant differences were found either in the exploratory group comparison of the volume of the entire amygdala (left *F* (3,75) = 0.48; right: *F* (3,76) = 1.58, all *p* >.05). Detailed statistics on group comparisons are displayed in **Table 3**.

In the ANCOVA examining the left whole amygdala and the left BLA, the assumption of homogeneity of regression slopes was violated when correcting for the covariates ICV and sex. This violation has little influence on alpha error probability or test power (93–96).

To compare patients with and without comorbid anxiety disorders in their medical history, as assessed with the SCID, we conducted Student’s *t*-tests, initially including all MDD patients, and subsequently analyzing MDDm and MDDu groups separately (see **Supplementary Material 1**). No statistically significant results were observed.

### 3.2 Correlation between numbers of MDEs or duration of illness and volumes of interest

The quadratic regressions between the number of MDEs and the volumes of the left and right BLA and LA, calculated for the pooled MDD group and for the MDDu and MDDm group, did not reach statistical significance (**Table 4**).

**Table 4:** Quadratic regressions on number of major depressive episodes and duration of illness.

		Left		Right	
		Basolateral Complex	Lateral Nucleus	Basolateral Complex	Lateral Nucleus
**MDD**	**Number of Major Depressive Episodes ~**	*R^2^* = .11, *F* (2, 37) = 2.21, *p* = .124	*R^2^* = .04, *F* (2, 37) = 0.85, *p* = .437	*R^2^* = .11, *F* (2, 37) = 2.30, *p* = .115	*R^2^* = .07, *F* (2, 37) = 1.45, *p* = .248
	**Duration of disease ~**	*R^2^* = .04, *F* (2, 36) = 0.82, *p* = .448	*R^2^* = .05, *F* (2, 36) = 1.01, *p* = .376	*R^2^* = .02, *F* (2, 36) = .43, *p* = .652	*R^2^* = .02, *F* (2, 36) = .45, *p* = .64
**MDDm**	**Number of Major Depressive Episodes ~**	*R^2^* = .13, *F* (2, 17) = 1.29, *p* = .300	*R^2^* = .05, *F* (2, 17) = 0.49, *p* = .622	*R^2^* = .16, *F* (2, 17) = 1.57, *p* = .236	*R^2^* = .11, *F* (2, 17) = 1.06, *p* = .369
	**Duration of disease ~**	*R^2^* = .01, *F* (2, 17) = 0.06, *p* = .945	*R^2^* = .01, *F* (2, 17) = 0.07, *p* = .933	*R^2^* = .00, *F* (2, 17) = 0.00, *p* = .998	*R^2^* = .00, *F* (2, 17) = 0.03, *p* = .975
**MDDu**	**Number of Major Depressive Episodes ~**	*R^2^* = .13, *F* (2, 17) = 1.25, *p* = .312	*R^2^* = .09, *F* (2, 17) = 0.85, *p* = .446	*R^2^* = .25, *F* (2, 17) = 2.86, *p* = .085	*R^2^* = .19, *F* (2, 17) = 2.03, *p* = .162
	**Duration of disease ~**	*R^2^* = .38, *F* (2, 16) = 4.87, *p* = .022*	*R^2^* = .36, *F* (2, 16) = 4.50, *p* = .028*	*R^2^* = .45, *F* (2, 16) = 6.42, *p* = .009**	*R^2^* = .40, *F* (2, 16) = 5.29, *p* = .017*

Coefficient of determination *R^2^*. *p ≤.05, **p ≤.01. *p* values are not corrected for multiple comparison using Bonferroni correction. MDD, Depressed patients; MDDm, Medicated depressed patients; MDDu, Unmedicated depressed patients.

**Figure 2:**
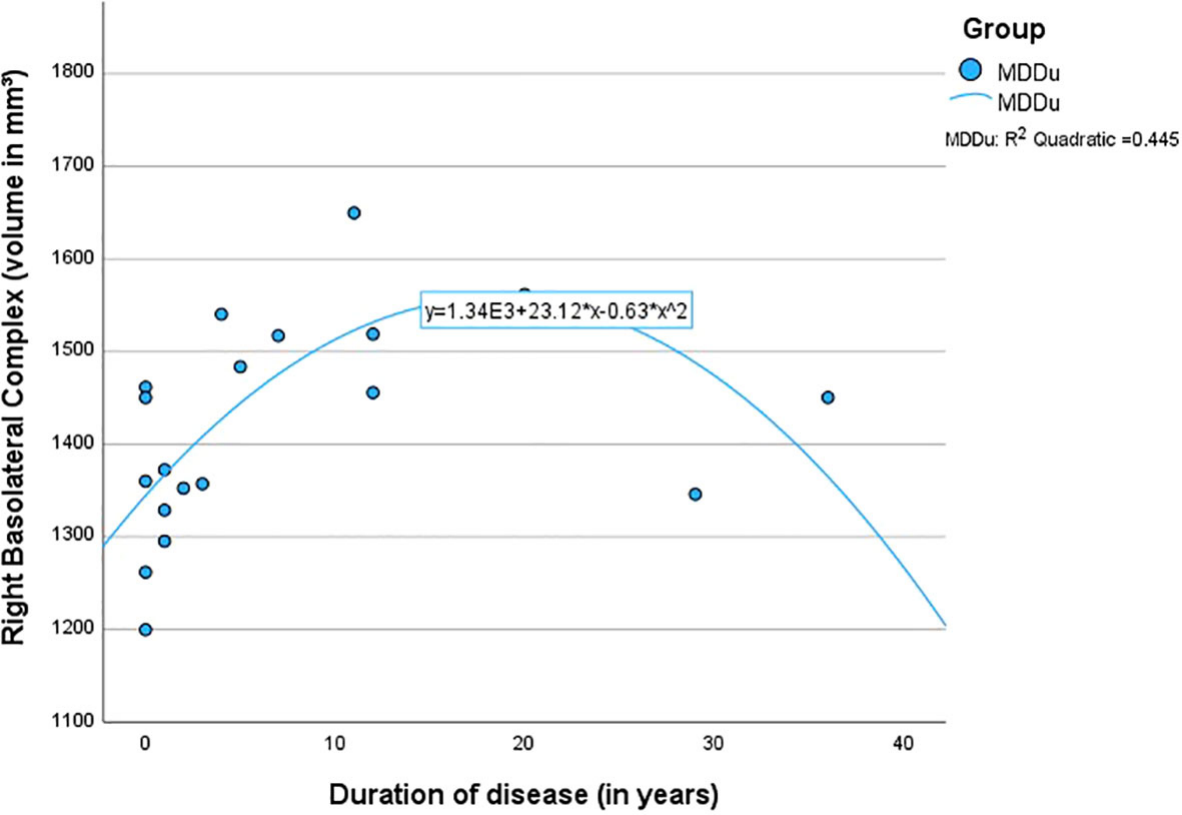
Quadratic regression between the duration of disease and the volume of the right basolateral complex.

The quadratic regressions between the overall duration since the onset of the first MDE and the volumes of the BLA (left *R^2^ = .*38, *p* <.05; right *R^2^ = .*45, *p* <.01) and LA (left *R^2^ = .*36, *p* <.05; right *R^2^ = .*40, *p* <.05) were found to be highly significant in the MDDu group. However, this effect was mainly driven by two individuals (duration 29 and 36 years; see **Figure 2**), limiting interpretability of the effect. None of the regressions in the other groups were found to be significant (**Table 4**).

### 3.3 Correlations with clinical characteristics

In the MDDm group, statistically significant correlations were observed between weeks since the onset of the current episode and the volumes of the left BLA (*ρ* = .49, *p* <.05), left LA (*ρ* = .47, *p* <.05) and right BLA (*ρ* = .47, *p* <.05). These correlations did not remain significant after adjusting for multiple comparisons using Bonferroni correction. None of the other correlations reached statistical significance. All clinical correlations are displayed in **Supplementary Material 2**.

## 4 Discussion

### 4.1 General considerations and reliability of the study

Our volume measurements obtained using 7T MRI align excellently with recent subfield studies (58, 60, 97) as well as with a meta-analysis (98), confirming the general validity of our volumetric estimates. The total and subfield volumes of the amygdala correlate bilaterally with the ICV, as anticipated (99). Previous studies (100) have reported that men tend to have larger amygdala volumes than women, a finding consistent with our results. However, this difference is likely due to variations in ICV rather than a true sexual dimorphism (99). The finding that age is not a confounder in our study is also consistent with existing literature (101, 102).

It is widely accepted that the amygdala exhibits subtle to moderate volumetric asymmetry in healthy individuals, typically favoring the right hemisphere (103, 104). This asymmetry is primarily attributed to a larger right-sided nucleus (97). In our sample, we observed slight asymmetries in favor of the right side (see asymmetry indices in **Supplementary Material 3**).

### 4.2 BLA/LA volumes (hypotheses I, II and III)

Contrary to our expectations, we did not find evidence of increased volumes in the BLA or the LA in the MDDu group compared to HC. Similarly, we did not find evidence of decreased volumes in the MDDm population.

The patients in the MDDm group had experienced more MDEs (*U*_40_ = 97.50, *p* = .005) and a longer period of time (*U*_39_ = 120.00, *p* = .050) had passed since their first episode (see **Table 1**). This suggests that their illness had progressed further compared to individuals from MDDu group. As previously evidenced, there is an augmentation in amygdala volume earlier in the progression of MDD (63, 69, 105), with a subsequent reduction as the disease advances (71).

However, the medication status of MDDm is also a potential confounder. Patients from the MDDm group were treated either with an antidepressant monotherapy, antidepressants combined with sedative drugs, or with a combination of antidepressants with lithium plus atypical neuroleptics. Several authors have proposed that antidepressant medication may influence amygdalar volumes (56, 106). The neuroprotective effects of antidepressant treatment may have offset or balanced the effects of cumulative glucocorticoid toxicity and excitotoxic damage. It is important to emphasize that both the unmedicated and medicated groups presented with markers of disease severity that did not differ statistically significantly. This suggests a persistent disease progression even under antidepressant medication. Therefore, it appears that the confounding effect could be considered manageable to a certain extent. To completely rule out this influence, two groups with the same or similar number of MDEs and duration of illness would have been necessary, differing only in medication status. However, this proved unattainable due to insufficient availability of eligible patients.

Our hypotheses proposed a trajectory wherein basolateral amygdala volumes would change from a baseline, as observed in healthy volunteers, to an enlarged state in individuals with MDDu (indicating a shorter duration of MDD), followed by a subsequent decline below the original baseline in individuals with MDDm (reflecting a longer course of the disease). However, establishing these baselines may be confounded by significant variations in both the shape and volume of the amygdala among individuals in the general population, a phenomenon influenced by factors such as genetics (107, 108). Consequently, the considerable inter-individual diversity in amygdala volumes within the normal spectrum might obscure the detection of effects specific to certain diseases. Detecting or ruling out minor changes in a variable with significant variation would therefore necessitate large sample sizes to ensure reliability.

Our finding of no significant group difference in BLA/LA volume is consistent with a recent subfield study by Brown et al. (62), which comprised a similar sample size (MDD *n* = 24). They used an older FreeSurfer version 6.0, and their MDD patients had been antidepressant free only for at least 4 weeks, while our MDDu sample was drug free for at least three months. Our results, indicating no significant group differences between patients with and without comorbid anxiety disorders, align with Li et al. (64). However, these findings should be interpreted with caution due to our small sample size (MDD with comorbid anxiety, *n* = 7).

### 4.3 No quadratic association between number of MDEs and the volumes of the BLA/LA (hypotheses IV and V)

Our second hypothesis of a negative quadratic association between the number of MDEs or duration of illness and the volumes of interest was also not supported. To our knowledge, negative quadratic associations between surrogate parameters of MDD progression and amygdalar volumes have not been tested before.

The number of MDEs in our study ranged between 1 and 5 (MDDu) and between 1 and 60 (MDDm). In MDDm group, however, the range was predominantly between 1 and 10 with two outliers at the count of 23 and 60 episodes. This range may have been insufficient for an inverted U-shape pattern to manifest.

By its very nature, attempting to investigate a time-dependent effect with a cross-sectional study design is very limited. Longitudinal studies on amygdalar volume alterations in MDD did not find changes in amygdala volume (109); however, they did not examine at the subnuclei level. To detect time-dependent changes in amygdalar volumes, large-scale longitudinal studies at the subnuclei level would be necessary.

The complexity and heterogeneity of MDD potentially constitute significant confounding factors, given the incompletely understood pathophysiology of the disease (3, 4). It is assumed that the different subtypes of MDD are based on varying underlying mechanisms (5, 6). E.g., a reduction in amygdala volume was observed in individuals with psychotic depression, but not in those with non-psychotic depression (110). Psychotic depression was rarely observed in our sample population; thus, it couldn’t be completely ruled out as a confounding factor. Cong et al. reported bilateral volume increases of the basal nuclei in their subfield study comprised of 59 first MDE patients with suicidal ideation (57). Enlarged amygdala volumes and suicidality have been associated before (111, 112). We did not assess for suicidal ideation in our sample. Early life adversity and psychological trauma need to be considered as well as potential powerful confounders. Both correlate with alterations in amygdala volumes (113) as well as with a heightened severity and chronicity of MDD (114) and diminished efficacy in treatment response and remission outcomes (115, 116). We did not take early life adversity and psychological trauma into account in this study.

### 4.4 Exploratory investigation of whole amygdala volume and clinical correlations

Our exploratory analysis of whole amygdala volumes did not reveal any significant group differences. This finding is consistent with several large meta-analyses, which did not identify significant changes in whole amygdala volumes. An exception is the study by Hamilton et al. (56) which included only medicated MDD patients. Given that some of the aforementioned subfield studies have reported volume increases in certain nuclei alongside decreases in others, it is likely that these effects counterbalance each other.

Our findings of no significant clinical correlations are consistent with those of the multicenter study by the ENIGMA group (65), and are also supported by the work of Roddy et al. (60) and Kim et al. (58). They stand in contrast to only two subfield studies. Brown et al. reported significant negative correlations between the severity of depressive symptoms and the volumes of the right LA, the right BLA and the left accessory basal nucleus (62). Tesen et al., in their study involving 76 drug-naïve individuals experiencing their first episode of MDD, identified an inverse linear correlation between the total and core scores of the Hamilton Rating Scale for Depression and the volume of the right LA (61).

### 4.5 Strengths and limitations

The utilization of a 7T MRI field strength offers superior signal quality and enables ultra-high resolution, which is particularly advantageous for distinguishing between the very small amygdala nuclei (117). We employed the highly up-to-date version 7.3.2 (August 2022) of FreeSurfer, which capitalizes on the submillimeter resolution of our images, thereby facilitating state-of-the-art automatic segmentation. An important advantage of automated segmentation methods such as ours is the reduction of investigator bias.

Our study comprised for the first-time uni- and bipolar depression *in vivo* cohorts that were carefully balanced for key confounding variables (age, sex, ICV, handedness, disease severity) and excluded individuals with neurological comorbidities. The initial diagnoses of MDD and BP were established through a structured interview cross-sectionally, and their accuracy was subsequently confirmed by longitudinal data provided by the treating psychiatrists. Our MDDu group refrained from psychotropic medication for a minimum of 3 months prior to the 7T MRI scan. Only original volumes were utilized for statistical analysis, and the results were rigorously adjusted by stepwise inclusion of all significant confounders.

A primary strength of this study lies in our meticulous hypothesis-driven approach. This sets it apart from some other recent studies on amygdalar subfield volumes, which have not explicitly stated their *a priori* hypotheses and have not provided compelling explanations for their findings. Consequently, we refrained from conducting tests on the other amygdala nuclei without establishing stringent hypotheses beforehand.

The sample sizes were calculated to ensure sufficient test power to detect large-sized effects. Given the aforementioned genetic variability of amygdala volumes, we acknowledge that the sample sizes (20 participants each in the medicated and unmedicated MDD groups) may have been too small to detect basolateral amygdala volume alterations. Larger sample sizes were not within the scope of this study, as recruiting unmedicated MDD patients proved to be very challenging.

We recognize that the more advanced MDD disease state of patients in the MDDm group is confounded by their medication status, making it harder to identify effects uniquely attributable to illness progression.

Despite the extensive validation efforts, there are also reasons to cautiously evaluate FreeSurfer’s results. Automated segmentation has significantly improved the feasibility of neuroimaging analysis and enables the processing of larger sample sizes within a reasonable timeframe. Nevertheless, there are recommendations for vigilance in interpreting the findings of these techniques (118). This is particularly true for “*the amygdala [which] is a highly complex structure with a small overall volume*” (97), making it notoriously difficult to measure accurately. Hanson et al., e.g., observed only low bivariate correlations between the automated amygdala segmentations generated by an older (2012) version of FreeSurfer and the volumes obtained from their hand-tracing (119).

### 4.6 Conclusions and implications for further research

In conclusion, this study could not confirm an inverted U-shaped trajectory of basolateral amygdala volumes during the course of MDD. To our knowledge, this is the first examination of the allostatic load theory in MDD concerning alterations in basolateral amygdala volume. No alternative theory to date has demonstrated comparable ability in integrating previously discordant findings on basolateral amygdala volume, while grounding them in robust biophysiological principles. We hope that other research groups will test the inverted U-shape hypothesis with larger sample sizes in the future.

To comprehensively assess the structural implications of affective disorders such as MDD on the brain, it will be advantageous to investigate not only clinical variables but also genetic and epigenetic characteristics. That approach may elucidate longitudinal volume alterations as the disease evolves, making it even more suitable to explore U-shaped trajectories.

## Data Availability

The raw data supporting the conclusions of this article will be made available by the authors, without undue reservation.
