# Supplementary material for: Basolateral amygdala volume in affective disorders using 7T MRI in vivo
Source: Front Psychiatry. 2025 Jan 6;15:1404594. doi: 10.3389/fpsyt.2024.1404594 (PMC11744004; doi:10.3389/fpsyt.2024.1404594)
Supplement: Supplementary file 1 [file Table1.docx]

Supplement 1. Comparison of MDD patients with and without anxious comorbidity

|  | **All MDD (*N* (anxious comorbidity) = 7)** | **MDDm (*N* (anxious comorbidity) = 5)** | **MDDu (*N* (anxious comorbidity) = 2)** |
| --- | --- | --- | --- |
| **Left Lateral Nucleus** | *t* (38) = 0.28, *p* = .783 | *t* (18) = 0.29, *p* = .772 | *t* (18) = - 0.06, *p* = .955 |
| **Left Basolateral Complex** | *t* (38) = 0.72, *p* = .477 | *t* (18) = 0.43, *p* = .674 | *t* (18) = 0.54, *p* = .599 |
| **Right Lateral Nucleus** | *t* (38) = - 0.18, *p* = .860 | *t* (18) = - 0.71, *p* = .485 | *t* (18) = 1.02, *p* = .323 |
| **Right Basolateral Complex** | *t* (38) = 0.30, *p* = .770 | *t* (18) = - 0.37, *p* = .719 | *t* (18) = 1.36, *p* = .192 |

Student’s *t*-Test. The *p* values listed in the table are not corrected for multiple testing using Bonferroni correction.
