# Supplementary material for: Basolateral amygdala volume in affective disorders using 7T MRI in vivo
Source: Front Psychiatry. 2025 Jan 6;15:1404594. doi: 10.3389/fpsyt.2024.1404594 (PMC11744004; doi:10.3389/fpsyt.2024.1404594)
Supplement: Supplementary file 2 [file Table2.docx]

Supplement 2. Correlations with clinical variables

| *Correlations for all patients* | | | | | | | | |
| --- | --- | --- | --- | --- | --- | --- | --- | --- |
|  | | | Left Whole Amygdala | Left Basolateral Complex | Left Lateral Nucleus | Right Whole Amygdala | Right Basolateral Complex | Right Lateral Nucleus |
| Spearman's rho | Age of Onset | Correlation Coefficient | -,063 | -,067 | -,044 | ,073 | ,079 | ,088 |
|  |  | Sig. (2-tailed) | ,640 | ,618 | ,743 | ,581 | ,554 | ,507 |
|  |  | N | 58 | 58 | 58 | 59 | 59 | 59 |
|  | Weeks since Onset current MDE | Correlation Coefficient | ,161 | ,197 | ,231 | ,172 | ,186 | ,213 |
|  |  | Sig. (2-tailed) | ,223 | ,135 | ,079 | ,190 | ,154 | ,102 |
|  |  | N | 59 | 59 | 59 | 60 | 60 | 60 |

| *Correlations for all patients* | | | | | | | | |
| --- | --- | --- | --- | --- | --- | --- | --- | --- |
|  | | | Left Whole Amygdala | Left Basolateral Complex | Left Lateral Nucleus | Right Whole Amygdala | Right Basolateral Complex | Right Lateral Nucleus |
| Kendall's tau | BDI | Correlation Coefficient | -,011 | -,016 | ,016 | ,028 | ,036 | ,093 |
|  |  | Sig. (2-tailed) | ,901 | ,860 | ,860 | ,754 | ,687 | ,298 |
|  |  | N | 59 | 59 | 59 | 60 | 60 | 60 |
|  | HRSD | Correlation Coefficient | -,003 | -,004 | -,017 | -,073 | -,053 | -,005 |
|  |  | Sig. (2-tailed) | ,974 | ,963 | ,849 | ,417 | ,553 | ,954 |
|  |  | N | 59 | 59 | 59 | 60 | 60 | 60 |
|  | BRMS | Correlation Coefficient | ,016 | ,013 | ,014 | -,038 | -,013 | ,039 |
|  |  | Sig. (2-tailed) | ,865 | ,885 | ,875 | ,673 | ,888 | ,664 |
|  |  | N | 59 | 59 | 59 | 60 | 60 | 60 |
|  | IDS | Correlation Coefficient | ,012 | ,016 | ,028 | -,005 | ,017 | ,054 |
|  |  | Sig. (2-tailed) | ,891 | ,860 | ,758 | ,959 | ,848 | ,548 |
|  |  | N | 59 | 59 | 59 | 60 | 60 | 60 |
|  | IDS Factor 2 | Correlation Coefficient | -,004 | ,002 | ,036 | -,035 | -,017 | ,002 |
|  |  | Sig. (2-tailed) | ,962 | ,984 | ,701 | ,703 | ,854 | ,979 |
|  |  | N | 58 | 58 | 58 | 59 | 59 | 59 |

MDE: Major depressive episode, BDI: Beck Depression Inventory, HRSD: Hamilton Rating Scale for Depression, BRMS: Bech-Rafaelsen-Melancholia Scale, IDS: Inventory of Depressive Symptomatology. The p values listed in the tables are not corrected for multiple testing using Bonferroni correction.

| *Correlations for all MDD patients* | | | | | | | | |
| --- | --- | --- | --- | --- | --- | --- | --- | --- |
|  | | | Left Whole Amygdala | Left Basolateral Complex | Left Lateral Nucleus | Right Whole Amygdala | Right Basolateral Complex | Right Lateral Nucleus |
| Spearman's rho | Age of Onset | Correlation Coefficient | -.084 | -.112 | -.109 | .046 | .058 | .085 |
|  |  | Sig. (2-tailed) | .609 | .499 | .507 | .779 | .726 | .607 |
|  |  | N | 39 | 39 | 39 | 39 | 39 | 39 |
|  | Weeks since Onset current MDE | Correlation Coefficient | .201 | .254 | .271 | .205 | .245 | .269 |
|  |  | Sig. (2-tailed) | .215 | .114 | .090 | .205 | .128 | .093 |
|  |  | N | 40 | 40 | 40 | 40 | 40 | 40 |

| *Correlations for all MDD patients* | | | | | | | | |
| --- | --- | --- | --- | --- | --- | --- | --- | --- |
|  | | | Left Whole Amygdala | Left Basolateral Complex | Left Lateral Nucleus | Right Whole Amygdala | Right Basolateral Complex | Right Lateral Nucleus |
| Kendall’s tau | BDI | Correlation Coefficient | ,000 | -,010 | ,029 | ,044 | ,042 | ,117 |
|  |  | Sig. (2-tailed) | 1,000 | ,926 | ,797 | ,692 | ,709 | ,294 |
|  |  | N | 40 | 40 | 40 | 40 | 40 | 40 |
|  | HRSD | Correlation Coefficient | ,047 | ,039 | ,036 | ,042 | ,078 | ,146 |
|  |  | Sig. (2-tailed) | ,674 | ,726 | ,744 | ,709 | ,484 | ,191 |
|  |  | N | 40 | 40 | 40 | 40 | 40 | 40 |
|  | BRMS | Correlation Coefficient | ,043 | ,038 | ,038 | ,025 | ,064 | ,156 |
|  |  | Sig. (2-tailed) | ,700 | ,735 | ,735 | ,824 | ,567 | ,164 |
|  |  | N | 40 | 40 | 40 | 40 | 40 | 40 |
|  | IDS | Correlation Coefficient | ,072 | ,079 | ,082 | ,090 | ,124 | ,173 |
|  |  | Sig. (2-tailed) | ,521 | ,476 | ,462 | ,420 | ,267 | ,120 |
|  |  | N | 40 | 40 | 40 | 40 | 40 | 40 |
|  | IDS Factor 2 | Correlation Coefficient | ,063 | ,074 | ,099 | ,074 | ,096 | ,135 |
|  |  | Sig. (2-tailed) | ,584 | ,519 | ,388 | ,519 | ,401 | ,238 |
|  |  | N | 39 | 39 | 39 | 39 | 39 | 39 |

MDE: Major depressive episode, BDI: Beck Depression Inventory, HRSD: Hamilton Rating Scale for Depression, BRMS: Bech-Rafaelsen-Melancholia Scale, IDS: Inventory of Depressive Symptomatology. The p values listed in the tables are not corrected for multiple testing using Bonferroni correction.

| *Correlations for medicated depressed patients (MDDm)* | | | | | | | | |
| --- | --- | --- | --- | --- | --- | --- | --- | --- |
|  | | | Left Whole Amygdala | Left Basolateral Complex | Left Lateral Nucleus | Right Whole Amygdala | Right Basolateral Complex | Right Lateral Nucleus |
| Spearman's rho | Age of Onset | Correlation Coefficient | -.131 | -.136 | -.137 | .090 | .089 | .058 |
|  |  | Sig. (2-tailed) | .582 | .566 | .564 | .707 | .709 | .808 |
|  |  | N | 20 | 20 | 20 | 20 | 20 | 20 |
|  | Weeks since Onset current MDE | Correlation Coefficient | .406 | .491 | .474 | .420 | .472 | .416 |
|  |  | Sig. (2-tailed) | .076 | .028* | .035* | .065 | .036* | .068 |
|  |  | N | 20 | 20 | 20 | 20 | 20 | 20 |

| *Correlations for medicated depressed patients (MDDm)* | | | | | | | | |
| --- | --- | --- | --- | --- | --- | --- | --- | --- |
|  | | | Left Whole Amygdala | Left Basolateral Complex | Left Lateral Nucleus | Right Whole Amygdala | Right Basolateral Complex | Right Lateral Nucleus |
| Kendall’s tau | BDI | Correlation Coefficient | -,123 | -,101 | -,059 | ,080 | ,133 | ,165 |
|  |  | Sig. (2-tailed) | ,454 | ,536 | ,720 | ,625 | ,416 | ,313 |
|  |  | N | 20 | 20 | 20 | 20 | 20 | 20 |
|  | HRSD | Correlation Coefficient | -,042 | -,021 | ,063 | ,095 | ,148 | ,116 |
|  |  | Sig. (2-tailed) | ,795 | ,897 | ,697 | ,559 | ,363 | ,475 |
|  |  | N | 20 | 20 | 20 | 20 | 20 | 20 |
|  | BRMS | Correlation Coefficient | -,032 | -,011 | ,053 | ,096 | ,149 | ,149 |
|  |  | Sig. (2-tailed) | ,845 | ,948 | ,745 | ,558 | ,363 | ,363 |
|  |  | N | 20 | 20 | 20 | 20 | 20 | 20 |
|  | IDS | Correlation Coefficient | -,100 | -,079 | -,016 | ,079 | ,111 | ,058 |
|  |  | Sig. (2-tailed) | ,537 | ,626 | ,922 | ,626 | ,495 | ,721 |
|  |  | N | 20 | 20 | 20 | 20 | 20 | 20 |
|  | IDS Factor 2 | Correlation Coefficient | ,136 | ,146 | ,233 | ,266 | ,298 | ,201 |
|  |  | Sig. (2-tailed) | ,414 | ,377 | ,160 | ,109 | ,072 | ,226 |
|  |  | N | 20 | 20 | 20 | 20 | 20 | 20 |
|  | | | | | | | | |

*p ≤ .05, MDE: Major depressive episode, BDI: Beck Depression Inventory, HRSD: Hamilton Rating Scale for Depression, BRMS: Bech-Rafaelsen-Melancholia Scale, IDS: Inventory of Depressive Symptomatology. The p values listed in the tables are not corrected for multiple testing using Bonferroni correction.

| *Correlations for unmedicated depressed patients (MDDu)* | | | | | | | | |
| --- | --- | --- | --- | --- | --- | --- | --- | --- |
|  | | | Left Whole Amygdala | Left Basolateral Complex | Left Lateral Nucleus | Right Whole Amygdala | Right Basolateral Complex | Right Lateral Nucleus |
| Spearman's rho | Age of Onset | Correlation Coefficient | -.099 | -.179 | -.215 | -.081 | -.064 | -.048 |
|  |  | Sig. (2-tailed) | .688 | .462 | .377 | .742 | .794 | .847 |
|  |  | N | 19 | 19 | 19 | 19 | 19 | 19 |
|  | Weeks since Onset current MDE | Correlation Coefficient | -.022 | .033 | .067 | .020 | .062 | .120 |
|  |  | Sig. (2-tailed) | .927 | .892 | .778 | .934 | .795 | .613 |
|  |  | N | 20 | 20 | 20 | 20 | 20 | 20 |

| *Correlations for unmedicated depressed patients (MDDu)* | | | | | | | | |
| --- | --- | --- | --- | --- | --- | --- | --- | --- |
|  | | | Left Whole Amygdala | Left Basolateral Complex | Left Lateral Nucleus | Right Whole Amygdala | Right Basolateral Complex | Right Lateral Nucleus |
| Kendall’s tau | BDI | Correlation Coefficient | ,154 | ,133 | ,122 | ,143 | ,133 | ,058 |
|  |  | Sig. (2-tailed) | ,346 | ,417 | ,455 | ,380 | ,417 | ,721 |
|  |  | N | 20 | 20 | 20 | 20 | 20 | 20 |
|  | HRSD | Correlation Coefficient | ,102 | ,092 | -,005 | -,038 | -,005 | ,038 |
|  |  | Sig. (2-tailed) | ,535 | ,579 | ,974 | ,819 | ,974 | ,819 |
|  |  | N | 20 | 20 | 20 | 20 | 20 | 20 |
|  | BRMS | Correlation Coefficient | ,147 | ,136 | ,049 | ,016 | ,049 | ,082 |
|  |  | Sig. (2-tailed) | ,377 | ,414 | ,769 | ,922 | ,769 | ,624 |
|  |  | N | 20 | 20 | 20 | 20 | 20 | 20 |
|  | IDS | Correlation Coefficient | ,272 | ,294 | ,218 | ,163 | ,196 | ,228 |
|  |  | Sig. (2-tailed) | ,102 | ,077 | ,190 | ,326 | ,238 | ,169 |
|  |  | N | 20 | 20 | 20 | 20 | 20 | 20 |
|  | IDS Factor 2 | Correlation Coefficient | ,000 | ,000 | -,037 | -,049 | -,073 | -,012 |
|  |  | Sig. (2-tailed) | 1,000 | 1,000 | ,832 | ,777 | ,671 | ,944 |
|  |  | N | 19 | 19 | 19 | 19 | 19 | 19 |
|  | | | | | | | | |

MDE: Major depressive episode, BDI: Beck Depression Inventory, HRSD: Hamilton Rating Scale for Depression, BRMS: Bech-Rafaelsen-Melancholia Scale, IDS: Inventory of Depressive Symptomatology. The p values listed in the tables are not corrected for multiple testing using Bonferroni correction.

| *Correlations for bipolar patients (BP)* | | | | | | | | |
| --- | --- | --- | --- | --- | --- | --- | --- | --- |
|  | | | Left Whole Amygdala | Left Basolateral Complex | Left Lateral Nucleus | Right Whole Amygdala | Right Basolateral Complex | Right Lateral Nucleus |
| Spearman's rho | Age of Onset | Correlation Coefficient | ,031 | ,049 | ,082 | ,099 | ,093 | ,043 |
|  |  | Sig. (2-tailed) | ,900 | ,843 | ,737 | ,679 | ,696 | ,856 |
|  |  | N | 19 | 19 | 19 | 20 | 20 | 20 |
|  | Weeks since Onset current MDE | Correlation Coefficient | ,059 | ,052 | ,119 | ,130 | ,087 | ,060 |
|  |  | Sig. (2-tailed) | ,811 | ,833 | ,626 | ,584 | ,717 | ,801 |
|  |  | N | 19 | 19 | 19 | 20 | 20 | 20 |

| *Correlations for bipolar patients (BP)* | | | | | | | | |
| --- | --- | --- | --- | --- | --- | --- | --- | --- |
|  | | | Left Whole Amygdala | Left Basolateral Complex | Left Lateral Nucleus | Right Whole Amygdala | Right Basolateral Complex | Right Lateral Nucleus |
| Kendall’s tau | BDI | Correlation Coefficient | -,059 | -,012 | ,000 | -,053 | -,032 | ,011 |
|  |  | Sig. (2-tailed) | ,726 | ,944 | 1,000 | ,745 | ,845 | ,948 |
|  |  | N | 19 | 19 | 19 | 20 | 20 | 20 |
|  | HRSD | Correlation Coefficient | -,041 | ,006 | -,030 | -,181 | -,181 | -,096 |
|  |  | Sig. (2-tailed) | ,806 | ,972 | ,861 | ,269 | ,269 | ,558 |
|  |  | N | 19 | 19 | 19 | 20 | 20 | 20 |
|  | BRMS | Correlation Coefficient | ,150 | ,186 | ,126 | -,027 | -,005 | ,081 |
|  |  | Sig. (2-tailed) | ,379 | ,275 | ,460 | ,870 | ,974 | ,625 |
|  |  | N | 19 | 19 | 19 | 20 | 20 | 20 |
|  | IDS | Correlation Coefficient | -,106 | -,059 | -,012 | -,143 | -,122 | -,037 |
|  |  | Sig. (2-tailed) | ,528 | ,726 | ,944 | ,380 | ,455 | ,820 |
|  |  | N | 19 | 19 | 19 | 20 | 20 | 20 |
|  | IDS Factor 2 | Correlation Coefficient | -,138 | -,090 | -,006 | -,226 | -,204 | -,140 |
|  |  | Sig. (2-tailed) | ,418 | ,598 | ,972 | ,171 | ,215 | ,397 |
|  |  | N | 19 | 19 | 19 | 20 | 20 | 20 |
| MDE: Major depressive episode, BDI: Beck Depression Inventory, HRSD: Hamilton Rating Scale for Depression, BRMS: Bech-Rafaelsen-Melancholia Scale, IDS: Inventory of Depressive Symptomatology. The p values listed in the tables are not corrected for multiple testing using Bonferroni correction. | | | | | | | | |
