# Supplementary material for: Basolateral amygdala volume in affective disorders using 7T MRI in vivo
Source: Front Psychiatry. 2025 Jan 6;15:1404594. doi: 10.3389/fpsyt.2024.1404594 (PMC11744004; doi:10.3389/fpsyt.2024.1404594)
Supplement: Supplementary file 3 [file Table3.docx]

Supplement 3. Asymmetry indices of amygdalar volumes for different groups

|  | **AI Whole Amygdala** | **AI Basolateral Complex** | **AI Lateral Nucleus** |
| --- | --- | --- | --- |
| **HC** | - 0.0101 | - 0.0048 | - 0.0001 |
| **MDDu** | - 0.0174 | - 0.0131 | - 0.0084 |
| **MDDm** | - 0.0098 | - 0.0029 | 0.0071 |
| **BP** | - 0.0067 | - 0.0017 | - 0.0007 |

AI: Asymmetry Index, calculated according to (115) as (Left volume – Right volume)/(Left volume + Right volume). Negative sign indicates that right-sided volumes are larger than left-sided. HC: Healthy controls, MDDu: unmedicated patients with major depressive disorder, MDDm: medicated patients with major depressive disorder, BP: patients with bipolar disorder.
